# Pathways Mediating the Interaction between Endothelial Progenitor Cells (EPCs) and Platelets

**DOI:** 10.1371/journal.pone.0095156

**Published:** 2014-06-05

**Authors:** Oshrat Raz, Dorit L. Lev, Alexander Battler, Eli I. Lev

**Affiliations:** 1 The Felsenstein Medical Research Institute, Petah-Tikva, Israel; 2 Cardiology Department, Rabin Medical Center, Jabotinsky St, Petah- Tikva, Israel; 3 Sackler Faculty of Medicine, Tel-Aviv University, Tel-Aviv, Israel; University of Torino, Italy

## Abstract

**Introduction:**

Endothelial progenitor cells (EPCs) have an important role in the process of vascular injury repair. Platelets have been shown to mediate EPC recruitment, maturation and differentiation. Yet, the mechanism underlying this interaction is unclear. We, therefore, aimed to examine whether direct contact between platelets and EPCs is essential for the positive platelets-EPC effect, and to investigate the main mediators responsible for the improvement in EPCs function.

**Methods:**

Human EPCs were isolated from donated buffy coats and cultured in either: 1. EPCs co-incubated with platelets placed in a 1 µm-Boyden chamber. 2. EPCs incubated with or without platelets in the presence or absence of bFGF/PDGF Receptor inhibitor (PDGFRI). After 7 days culture, EPCs ability to form colonies, proliferate and differentiate was examined. Culture supernatants were collected and growth factors levels were evaluated using ELISA. Growth factors mRNA levels in EPCs were evaluated using RT-PCR.

**Results and Conclusions:**

After 7 days culture, EPCs functional properties were higher following co-incubation with platelets (directly or indirectly), implying that direct contact is not essential for the platelet’s positive effect on EPCs. This effect was reduced by PDGFRI inhibition. Additionally, higher levels of PDGFB in EPCs-platelets supernatant and higher levels of PDGFC mRNA in EPCs co-incubated with platelets were found. In contrast, FGF and other potential mediators that were examined and inhibited did not significantly affect the interaction between platelets and EPCs. Thus, we conclude that PDGF has a central role in the interaction between platelets and EPCs. Further study is required to examine additional aspects of EPC-platelets interaction.

## Introduction

Endothelial Progenitor Cells (EPCs), are present in the circulation as peripheral blood mononuclear cells (PBMNCs) and exhibit phenotypic features of myeloid and endothelial cells [1], [2]. EPCs co-express CD133, CD34 and vascular endothelial growth factor receptor 2 (VEGFR-2) on their surface and have the potential to proliferate and differentiate into mature cells with endothelial phenotypic markers [3], [4], [5].

Previous studies have suggested that these cells participate in the process of neovascularization and re-endothelialization following vascular injury [2], [4], [6], [7], [8], [9], [10], [11]. It has been reported that following vascular injury or ischemia these cells are recruited to the site of injury and enhance neovascularization and re-endothelization [3], [4], [5], [10] Furthermore, circulating CD34+VEGFR2+ progenitor cells appear to have prognostic importance and their levels predict the occurrence of cardiovascular events including mortality in patients with cardiovascular disease [12]. In addition, we and others have shown a significant correlation between various cardiovascular risk factors and cardiovascular disease states and attenuated EPCs level and function. EPCs isolated from patients with coronary artery disease, for instance, have a reduced capacity to migrate, proliferate, and form colonies and to differentiate [13], [14], [15], [16], [17], [18].

The role of EPCs in vascular injury repair has been shown to involve an interaction with platelets. Several studies have indicated that platelets play an important role in the recruitment of EPCs to sites of vascular injury, and in their maturation and differentiation.[19], [20], [21], [22], [23]. *In vitro,* a significant interaction occurs between EPCs and activated platelets under both static and flow conditions [20], [21], [24]. These observations have gained support from *in vivo* experiments of carotid injury in mice which have demonstrated that platelets provide a critical signal for the early recruitment of bone marrow-derived progenitor cells, such as CD34+ cells, to the sites of vascular injury [23]. Apart from this effect, platelets appear to support and promote the maturation and differentiation of EPCs to cells expressing endothelial markers and to augment their functional properties [19], [22]. Exposure to platelets in culture conditions enhances the capacity of EPCs to form colonies, proliferate, migrate, express endothelial markers and produce NO metabolites (reflecting eNOS activity) [19], [22].

Although the effect of platelets on EPCs and their differentiation into cells with endothelial markers has been well-documented, the mechanism of this interaction remains unclear. One possible explanation is that the positive effect of platelets on EPCs functional properties may be mediated by various growth factors and chemokines that are secreted by platelets, such as platelet derived growth factor (PDGF) and basic fibroblast growth factor (bFGF). Both PDGF and bFGF are key factors in the angiogenic process [25], [26]. Platelet derived growth factor B (PDGFB) and platelet derived growth factor C (PDGFC) isoforms are essential for blood vessel maturation and have been shown to stimulate the recruitment of EPCs from the bone marrow and promote their differentiation into cells with endothelial or smooth muscle cells markers [26], [27]. FGF accelerates survival, proliferation and migration of endothelial cells [25]. Furthermore, it has been shown that FGF promotes the proliferation and migration of EPCs [28]. Therefore, the aims of this study were: 1. to evaluate whether direct contact between EPCs and platelets is necessary for the improvement of EPCs functional properties. 2. To investigate the role of potential mediators such as PDGF and FGF in the interaction between platelets and EPCs.

## Materials and Methods

### 1. Isolation of EPCs

Human early EPCs (eEPCs) were isolated from a Buffy coat, donated from an anonymous, single, healthy volunteer aged between 20–60. Mononuclear cells (PBMCs) were isolated by Ficoll density-gradient centrifugation. A total of 2×10^6^ PBMCs/ml were placed on 24 cm culture dishes coated with fibronectin and maintained in m-199 medium supplemented with 10% FBS for 5 to 7 days. [2]. After 7 days, PBMCs that had been cultured under these specific conditions developed a spindle-shaped appearance, formed typical cell clusters and took up acetylated LDL (Biomedical Technologies Stoughton MA, USA). These cells were considered as eEPCs.

### 2. Isolation of Platelets

Peripheral venous blood was drawn from healthy anonymous volunteers (aged 30–60 years), and collected in heparinized tubes. After centrifugation at 130 *g* for 20 min, platelet-rich plasma was removed, added to 400 u/ml heparin, and centrifuged at 900 *g* for 10 min. After removal of the supernatant, the resulting platelet pellet was washed twice in phosphate buffer saline (PBS) containing 400 u heparin/ml and re-suspended in endothelial medium without any supplement [13].

Blood was collected using a protocol approved by the Institutional Review Board for Human Subject Research (Helsinky Committee) at the Rabin Medical Center, Petah Tikva, (Israel). These blood products were donated by anonymous, healthy volunteers to the blood bank. Thus, no informed consent was obtained for our specific study (consent was given for the blood donation).

### 3. EPCs and Platelets Incubation

#### 3.1. Indirect co incubation EPCs and platelets

EPCs were co-incubated indirectly with washed platelets that were placed in a 1 µm Boyden chamber for 5–7days.

#### 3.2. Growth factors inhibition

Human EPCs were incubated alone or with platelets in the presence or absence of 2 ug/ml FGF basic Antibody (R&D, Minneapolis, USA) or 12.5 ug/ml PDGFR Tyrosine Kinase Inhibitor III (Santa Cruz, Ca, USA. This concentration was determined by dose-response experiments using several concentrations). Growth factors inhibitors were added to platelets and/or EPCs for 30 minutes prior incubation.

### 4. Assay of Colony Forming Units

EPC colonies were counted using an inverted microscope 5–7 days after plating on fibronectin-coated wells with or without platelets in the presence or absence of different inhibitors. An EPC colony was defined as a cluster of at least 100 flat cells surrounding a cluster of rounded cells. A central cluster alone without associated emerging cells was not counted as a colony [22]. For each test, colonies were counted by a double blinded observer in 10 different random fields. In order to confirm endothelial cell lineage, indirect immunostaining of randomly selected colonies against Tie-2, CD31 (Becton Dickinson, NJ, USA) was performed. The final results are expressed as the mean number of CFUs per field of all experiments [22].

### 5. Flow Cytometry Analysis for Endothelial Cell Markers

After 5–7 days in culture the cells were detached using Trypsin EDTA, centrifuged and re-suspended in 100 ul PBS. Aliquots of EPCs were incubated with monoclonal antibodies against Tie-2, CD31 (FITC labelled) (Santa Cruz, Califirnia, USA), VE-Cadherin (PE-labelled) (Santa Cruz, Ca, USA) and VEGFRII (FITC labeled) (R&D, Minneapolis, USA). Isotype-identical antibodies were used as controls. After incubation, cells were washed with phosphate-buffered saline and analyzed with a flow cytometer (FACS Calibur, Becton Dickinson). Each analysis included 10000 events, after the exclusion of debris. Analyses were performed in duplicates. Results are presented as the percentage of EPCs positive for the tested endothelial marker [29].

### 6. MTT Assay

The MTT assay measures mitochondrial activity in living cells and enable to estimate the viability of the cultured EPCs. MTT (3-[4,5-dimethylthiazol-2-yl]-2,5-diphenyl tetrazolium bromide) (Sigma, St.Louis, USA) 1 mg/ml was added to the EPC medium culture, after 5–7 days incubation (with or without platelets) and incubated for an additional 3–4 h. After incubation, the medium was removed and the cells were solubilized in isopropanol. The amount of the dye released from the cells was measured with a spectrophotometer at 570 nm and subtracted background at 690 nm. [22].

### 7. ELISA

After 5–7 days of EPCs and platelets co incubation, culture media was collected and measured for PDGF and FGF levels using Quansys multi ELISA kit (Quansys Biosciences, Logan, Utah, USA) according to the manufacturer’s protocol.

### 8. Quantitative Real Time PCR (RT-PCR)

After 7 days of culture with or without platelets, total RNA was purified from EPCs using TRIzol (Ambion, USA) according to the manufacturer’s instructions. The quantity of total RNA was determined by OD260 measurements. cDNA was synthesized from total RNA using the TaqMan High Capacity cDNA Reverse Transcription Kit (Applied Biosystems, Foster City, CA, USA) according to the manufacturer’s protocol. Quantitative real-time PCR analysis (Taqman) for PDGFB, PGDFC and FGF mRNA levels was performed using the StepOnePlus. Real-Time PCR System (Applied Biosystems; Foster City, CA, USA). A total of 3 µl of cDNA was amplified with 5 µl 2×TaqMan Gene Expression Master Mix, 0.5 µl 20×TaqMan Gene Expression Assay for PDGFB, PDGFC, FGF-2 and actin B (Applied Biosystems; Foster City, CA, USA), 1.5 µL DEPC. PCR amplification was performed consisting of 2 min at 50°C, 20 sec at 95°C, one cycle of 1 sec at 95° and 40 cycles of 20 sec at 60°C. All the samples were normalized to an endogenous gene, human β actin [29], [30].

### 9. Statistical Analysis

EPC parameters (results of the functional assays, flow cytometry determined levels, protein and mRNA levels) are presented as mean ± standard error (SE). Since EPCs parameters are non-normally distributed [15] the comparisons between the three groups were performed by Friedman tests followed by Wilcoxon matched-pairs signed-rank tests, two-tailed tests. Analyses were performed using SPSS version 15 statistical software (SPSS Inc., Chicago, IL, USA), and P = 0.05 was considered statistically significant.

## Results

### 1. EPCs Identification

**Figure 1:**
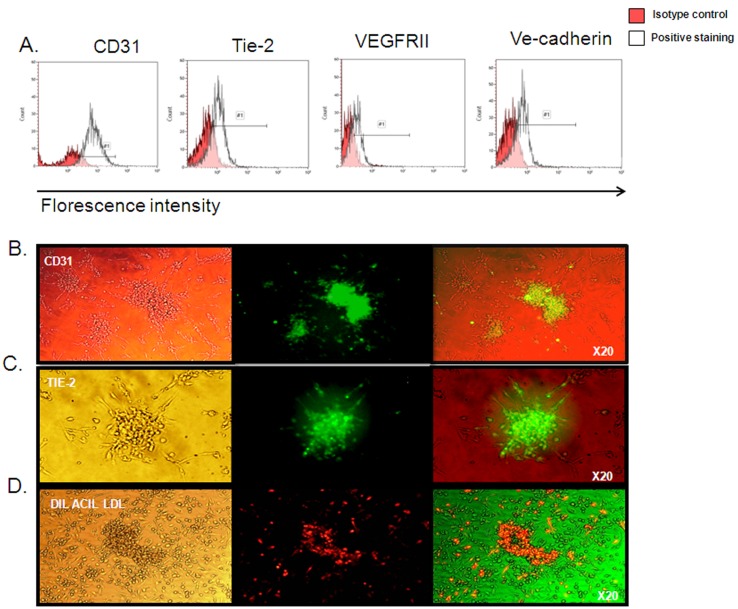
EPCs characterization. A. Representative FACs analysis figures for CD31, Tie-2, VEGFRII and VE-cadherin positive staining in EPCs after 7 days in culture. B–C. Positive immunohistochemical staining of the endothelial markers CD31 (C) and Tie-2 (D) in EPCs colonies. D. Positive uptake of CIL ACIL LDL in EPCs colonies.

To identify EPCs, Dil-Acil-LDL engulfment, and the endothelial lineage markers CD31, Tie-2 VE-cadherin and VEGFRII expression were measured. After 7 days of culture, positive staining was observed for all endothelial cell markers, as seen by FACs analysis (Figure 1A). In addition, all colonies stained positive for all the tested markers (e.g. CD31, Tie-2 and Dil-Acil-LDL engulfment, Figure 1B–D).

### 2. Indirect and Direct Incubation of EPCs and Platelets

**Figure 2:**
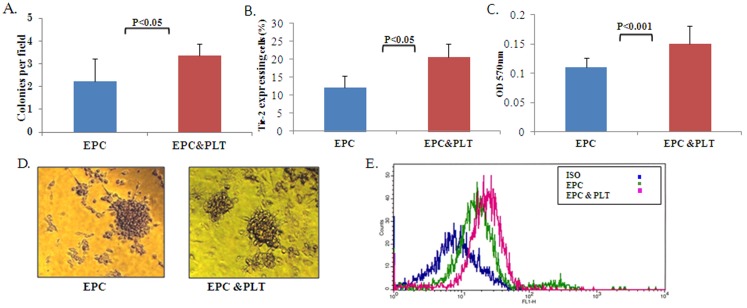
The indirect effect of platelets on EPCs’ functional properties. **A.** The average number of colonies per field ± SE in EPCs cultured with vs. without platelets, p<0.05, n = 13 (Wilcoxon matched-pairs signed-rank test). The capacity to form colonies was higher in EPCs cultured with platelets compared to EPCs cultured alone**. B.** Culture viability expressed as OD (560 nm) ± SE in EPCs cultured with vs. without platelets, p<0.05, n = 11 (Wilcoxon matched-pairs signed-rank test). EPCs cultured with platelets have enhanced culture viability. **C.** FACS analysis of the average number of Tie-2 expressing cells ± SE in EPCs cultured with vs. without platelets, p<0.05, n = 7 (Wilcoxon matched-pairs signed-rank test. A higher percent of Tie-2 expressing cells appear in EPCs cultured with platelets compared to EPCs cultured alone. **D.** Representative EPC colonies with platelets (right) compared to EPCs cultured alone (left). **E.** FACS analysis representative figure of Tie-2 expressing cells.

**Figure 3:**
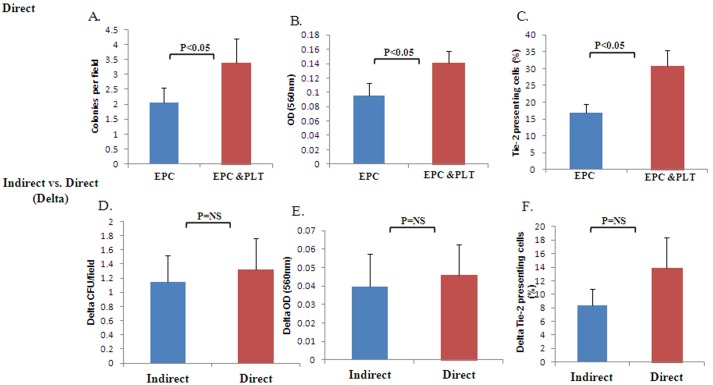
The direct effect of platelets on EPCs functional properties and its comparison to the platelets’ indirect effect. **A.** The average number of colonies per field ± SE in EPCs cultured with vs. without platelets, p<0.05, n = 13 (Wilcoxon matched-pairs signed-rank test). The capacity to form colonies was higher in EPCs cultured with platelets compared to EPCs cultured alone. **B.** Culture viability expressed as OD (560 nm) ± SE in EPCs cultured with vs. without platelets, p<0.05, n = 11 (Wilcoxon matched-pairs signed-rank test). EPCs cultured with platelets have enhanced culture viability. **C.** FACS analysis of the average number of Tie-2 expressing cells ± SE in EPCs cultured with vs. without platelets, p<0.05, n = 7 (Wilcoxon matched-pairs signed-rank test. A higher percent of Tie-2 expressing cells appear in EPCs cultured with platelets compared to EPCs cultured alone. D–F Direct vs indirect effect of platelets on EPCs ability to form colonies (D), culture viability (E) and the expression of Tie-2(F), for n = 13 or 11 or 7 respectively, p = NS for all. There was no significant difference in any of the tested parameters in EPCs incubated with platelets directly vs. indirectly.

In order to examine whether direct interaction between platelet and EPCs is essential for improving EPCs functional properties, we co-incubated the mononuclear cells indirectly with washed platelets that were placed in a 1 µm Boyden chamber or directly with washed platelets on the same plate. We investigated EPCs ability to form colonies, culture viability and the expression of Tie-2 after 7 days of culture. We found that the number of colonies and culture viability was significantly higher in EPCs that had been co-incubated with platelets directly or indirectly compared to EPCs that were incubated on fibronectin alone [for indirect incubation: 3.11±0.48 vs. 2.22±0.35 colonies per field, respectively (Figure 2A, 2C) and 0.15±0.031 vs. 0.11±0.016 OD 560 nm, respectively (Figure 2B), for direct incubation: 3.37±0.81 vs. 2.05±0.48 colonies per field respectively (Figure 3A) and 0.141±0.025 vs. 0.095±0.0172 560 nm, respectively (Figure 3B)]. Furthermore, EPCs that were co-incubated with platelets both directly or indirectly, had a higher expression of Tie-2 compared to EPCs that were incubated alone (20.56±3.72% vs. 12.23±3.14% cells expressed Tie-2 respectively for indirect incubation and 30.8±5% vs. 16.93±5% cells expressed Tie-2 for direct incubation, Figure 2C, 2E). Moreover, there was no significant difference between the direct vs. indirect effect of platelets on all EPCs functional properties tested (The platelets’ effect was calculated as the difference in EPCs function between EPCs cultured with platelets compared to EPCs cultured alone) (Figure 3D–E).

### 3. Inhibition of Potential Mediators and EPCs Functional Properties

To investigate the potential role of FGF and PDGF in the interaction between platelets and EPCs, mononuclear cells (MNC) and platelets were incubated with human FGF basic antibody or only MNC with PDGFR Tyrosine Kinase Inhibitor. After 7 days of culture EPCs ability to form colonies, cultured viability and differentiation capacity were investigated.

**Figure 4:**
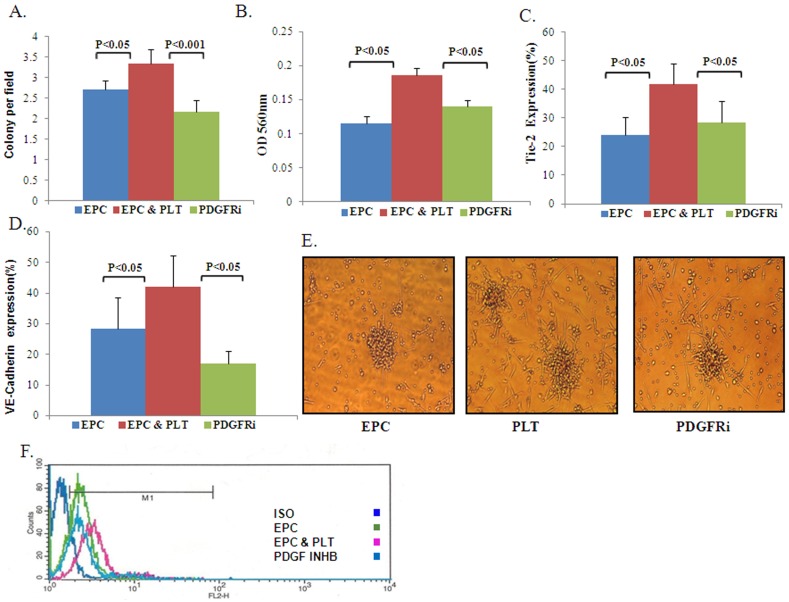
PDGF inhibition and its effect on EPCs functional properties. **A.** The average number of colonies per field ± SE in EPCs cultured with platelets compared to EPCs cultured with platelets and PDGF inhibitor or alone, n = 23, p<0.05, p<0.001. (Friedman test followed by Wilcoxon matched-pairs signed-rank test) The ability to form colonies was higher in EPCs cultured with platelets compared to EPCs cultured alone or with platelets and PDGF inhibitor. **B.** Culture viability expressed as OD (560 nm) ± SE in EPCs cultured with platelets compared to EPCs cultured with platelets and PDGF inhibitor or alone p<0.05, n = 11(Friedman test followed by Wilcoxon matched-pairs signed-rank test). EPCs cultured with platelets had greater culture viability compared to EPCs cultured with platelets and PDGF inhibitor or alone**. C–D** FACS analysis expressed as the average number of VE-cadherin (C) and Tie-2(D) expressing cells ± SE in EPCs cultured with platelets compared to EPCs cultured with platelets and PDGF inhibition or alone, p<0.05, n = 8 for C, p<0.05, n = 11 for D (Friedman test followed by Wilcoxon matched-pairs signed-rank test)**.** EPCs cultured with platelets have a higher percent of VE-cadherin (C) and Tie-2(D) expressing cells compared to EPCs cultured alone or with platelets and PDGF inhibitor. **E.** Representative EPC colonies with platelets (center) compared to EPCs cultured alone (left) or with platelets and PDGF inhibitor (right). **F.** FACs analysis representative figure of VE-cadherin expressing cells in EPCs cultured with platelets with or without PDGF inhibitor.

We found that EPCs functional properties were higher in EPCs co-incubated with platelets compared to those cultured alone. The platelets’ positive effect on EPCs was attenuated by the PDGFR Tyrosine Kinase Inhibitor: (1) EPCs capacity to form colonies [EPCs: 2.71±0.28; EPCs+platelets: 3.6±0.51; PDGFRI: 2.46±0.41 colonies per field (Figure 4A, 4E)]; (2) culture viability [EPCs: 0.115±0.01; EPCs+platelets: 0.186±0.01; PDGFRI: 0.140±0.009 OD 560 nm (Figure 4B)] and (3) endothelial markers: [EPCs: 23.94±6.32% EPCs+platelets: 41.81±7.046%; PDGFRI: 28.25±7.61% cells expressed Tie-2 (Figure 4C) and EPCs: 28.27±10.25%; EPCs+platelets: 42.19±10.09%; PDGFRI: 16.96±4.19% cells expressed VE-cadherin (Figure 4D, 4F)].

**Figure 5:**
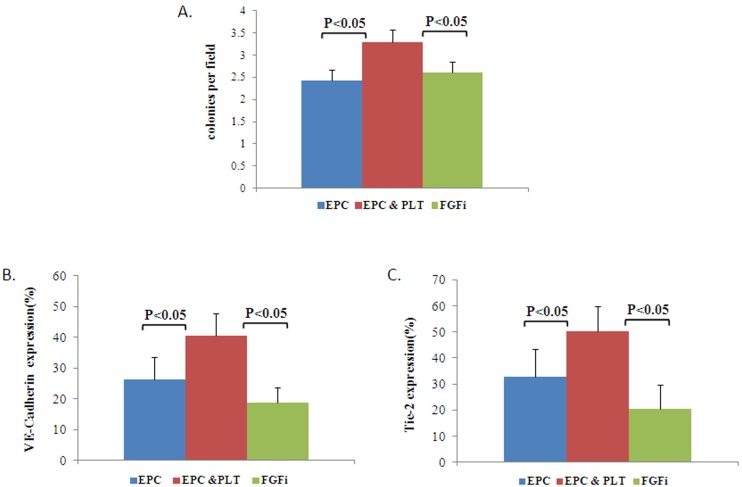
bFGF inhibition and its effect on EPCs differentiation. **A.** The average number of colonies per field ± SE in EPCs cultured with platelets compared to EPCs cultured with platelets and FGF inhibitor or alone, P<0.05, n = 23 (Friedman test followed by Wilcoxon matched-pairs signed-rank test) The ability to form colonies was higher in EPCs cultured with platelets compared to EPCs cultured alone or with platelets and FGF inhibitor. **B–C.** FACS analysis of the average number of VE-cadherin (A) and Tie-2 (B) expressing cells ± SE, p<0.05, n = 11 for A, p<0.05 n = 10 for B. EPCs cultured with platelets have a higher proportion of Tie-2 and VE-cadherin expressing cells compared to EPCs cultured alone or with platelets and bFGF inhibitor.

FGF inhibition attenuated only part of the positive effects platelets exert on EPCs. FGF inhibition reduced the capacity of EPCs to form colonies [EPCs: 2.43±0.22; EPCs+platelets: 3.3±0.28; FGFI: 2.6±0.22 colonies per field, Figure 5A] and to express the endothelial cells markers - VE-cadherin and Tie-2 [EPCs: 26.38±7.21%; EPCs+platelets: 40.42±7.23%; FGFI: 18.72±5.03% cells expressed VE-cadherin, (Figure 5B) and EPCs: 32.73±10.64%; EPCs+platelets: 50.06±9.85%; FGFI: 20.36±9.45% cells expressed Tie-2 (Figure 5C)]. However, no significant difference was observed in culture viability when EPCs were cultured with platelets compared to EPCs that were cultured with platelets and bFGF inhibitor or alone (data not shown).

### 4. PDGF and FGF Protein Levels in EPCs-platelet (PLT) Culture Media and Relative mRNA Expression in EPCs

**Figure 6:**
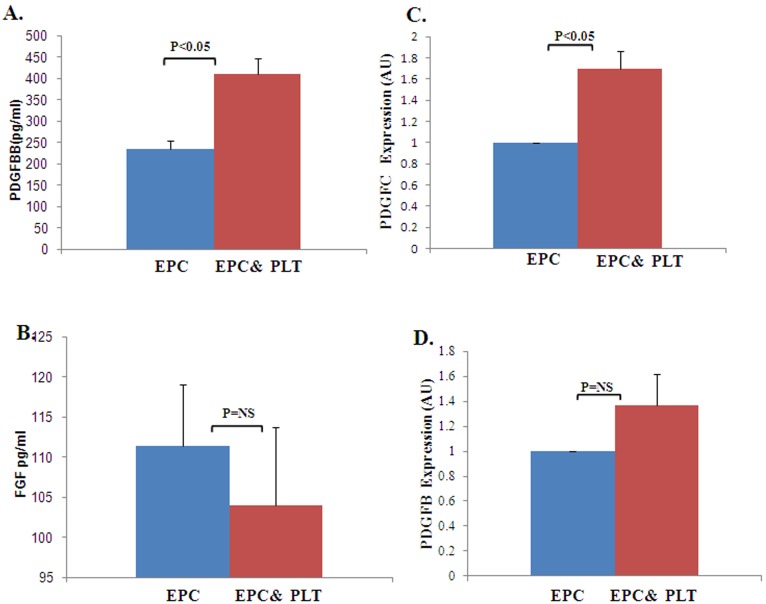
PDGF and FGF protein levels on EPC-PLT supernatant and relative PDGF B/C mRNA levels in EPCs following co incubation with platelets. **A–B.** PDGF(A) and FGF(B) levels expressed as pg/ml ± SE in supernatants of EPCs cultured with vs. without platelets, n = 11, p<0.05 for A, n = 5 p = NS for B (Wilcoxon matched-pairs signed-rank test). PDGF levels were significantly higher in EPCs cultured with platelets compared to EPCs cultured alone. There were no significant differences in FGF levels between the two groups. **C–D.** PDGFC(C) and PDGFB(D) relative expression appears as AU ± SE in EPCs cultured with vs. without platelets, n = 6 p<0.05 for A, n = 8, p = NS for B (Wilcoxon matched-pairs signed-rank test). PDGFC mRNA levels were significantly higher in EPCs cultured with platelets compared to EPCs cultured alone (C). There were no significant differences in PDGFB levels between the two groups (D).

In order to further examine the role of PDGF and bFGF in the interaction between EPCs and platelets we measured their levels in EPCs-platelets cells culture media using ELISA. We found that PDGFB levels were significantly higher in EPCs that were cultured with platelets compared to EPCs that were cultured alone (409.5±38.37 pg/ml vs. 234.5±67.76 pg/ml respectively, Figure 6A). However, there was no significant difference in bFGF levels (103.936±9.84 pg/ml vs. 111.372±7.66 pg/ml respectively, figure 6B). Our next step was to examine whether either platelets or EPCs secrete these growth factors. Accordingly, we measured PDGFB, PDGFC and FGF-2 mRNA transcript levels in EPCs cultured with or without platelets using Quantitative RT-PCR. We found a higher expression of PDGFC mRNA transcripts in EPCs that were cultured with platelets compared to EPCs that were cultured alone (1.7±0.17 vs. 1AU respectively, Figure 6C). PDGFB mRNA expression also appeared to be numerically higher in the EPCs cultured with platelets vs. EPCs alone. However, the differences were not significant (1.4±0.25 VS 1 AU respectively, P = NS, Figure 6D). In contrast, a very low expression of FGF mRNA was found in both groups (EPCs cultured alone or with platelets), with no differences between the groups (data not shown).

## Discussion

This study examined the potential pathways mediating the interaction between EPCs and platelets, and the positive effect platelets exert on EPCs. We found that direct contact is not essential for the effect platelets exert on EPCs, and that indirect co-incubation of EPCs and platelets had a beneficial effect on EPCs function. In addition, we found that PDGF has a central role in mediating the EPCs-platelets interaction.

Previous studies have indicated that EPCs have a central role in the process of vascular injury repair and that platelets mediate their recruitment to the site of injury. Moreover, it has been reported that *in-vitro* platelets enhance EPCs capacity to form colonies, proliferate, migrate and express endothelial markers [5], [19], [20], [22], [23], [24]. Furthermore, platelet microparticles have been shown to enhance the vasoregenerative potential of EPCs after vascular injury [29]. In this study we have shown that indirect co incubation of EPCs and platelets improved EPCs ability to form colonies, proliferate and express endothelial markers. We also found that there was no significant difference in the effect platelets exerts on EPCs when incubated directly vs. indirectly. Thus, it appears that the positive effect of platelets on EPCs does not require contact and that platelets products such as microparticles and secreted factors generate a microenvironment which is essential for this interaction.

Based on these results, we hypothesized that the improvement in EPCs function may be influenced by secreted factors originating from platelets or from EPCs as a response to platelets. In order to investigate the role of secreted factors, we examined the proangiogenic factors PDGF and FGF that are known to be key elements in the angiogenesis process [25], [26]. We found that PDGFRII inhibition attenuates the ability of platelets to enhance the formation of colonies, proliferation and expression of the endothelial markers TIE-2 and VE-cadherin on EPCs. Furthermore, there was a higher concentration of PDGFBB in the EPCs-platelets growth media compared to EPCs growth media. However, there was no significant increase in PDGFB mRNA levels in EPCs co-incubated with platelets compared with controls.

The discrepancy between the effect of platelets on PDGFB levels in growth culture media and PDGFB mRNA levels in EPCs can be explained by the fact that platelets are a major source of PDGF. Thus, higher levels of PDGFB in culture supernatant may result primarily from platelet secretion. Accordingly, EPCs may produce PDGFB independently of the effect of platelets. These results may also be explained by the lapse in time between mRNA transcription and protein assembly. It is possible that at this stage there is a significant difference in the protein level and not in mRNA levels since the mRNA transcripts have already been degraded. Accordingly the difference in protein levels in the supernatants might result from the secretion of both platelets and EPCs. Further study is required in order to clarify the source of PDGFB elevation in the EPCs-platelets supernatant.

Interestingly, when we measured the levels of PDGFC mRNA transcripts, we found that after incubation with platelets the expression of PDGFC mRNA transcripts was significantly higher. These results may indicate that platelets not only secrete this factor but also accelerate its secretion by EPCs. Taken together our results amplify the significance of PDGFB and PDGFC isoforms to EPCs-platelets interaction and to EPCs function generally.

Several prior studies support the ability of PDGF isoforms to promote EPCs functional properties. PDGFBB was reported to enhance EPCs ability to migrate and proliferate, and PDGFCC was shown to improve EPCs recruitment from the bone marrow and their differentiation into cells expressing endothelial markers [27], [28]. Thus, PDGF appears to have a beneficial effect on EPCs and a central role in the improvement in EPCs functional properties in response to platelets.

In contrast to PDGF, FGF inhibition did not attenuate the effect of platelets on EPCs in several of the functional assays. Additionally, exposure EPCs to platelets in culture did not affect the levels of FGF in the culture growth media. Furthermore, low levels of FGF mRNA were observed in EPCs with or without platelets, with no significant differences between them. On the other hand, FGF inhibition attenuated EPCs ability to form colonies and express the endothelial markers Tie-2 and VE-cadherin in response to platelets. Previous studies have indicated that FGF is essential for the acceleration of angiogenesis by platelets. Inhibition of FGF has been reported to attenuate platelets’ and platelet microparticles’ ability to enhance angiogenesis in a rat aortic ring model and to increase cell proliferation and survival of cultured endothelial cells [31], [32], [33]. Taken together, these findings indicate that FGF has a partial effect on EPCs which may include enhancement of an endothelial phenotype and the expression of endothelial cell markers. Furthermore, our findings imply that this effect is generated by primary secretion of FGF from platelets, rather than secretion from EPCs.

Previous studies have indicated that early EPCs have the ability to secret a wide range of mediators that are able to affect angiogenesis and vascular repair processes by an autocrine manner [29], [34], [35]. It is possible that the effect of PDGF and FGF which was observed in our study was influenced by other mediators and combined signal transduction pathways. It has already been shown that PDGFBB and bFGF have a synergistic effect on the proliferation and migration of EPCs as well as VEGF release from the cells. It has also been reported that bFGF promote proliferation and migration of EPCs by triggering PDGF Receptor β [28], [36]. Accordingly it is possible that bFGF and PDGF can work synergistically in the acceleration of a common signal pathway and enhance VEGF release to improve EPCs function. This subject should be further investigated in order to reveal the signal transduction pathways involved.

The effect platelets exert on EPCs in the direct co-culture conditions has been well established [13], [22]. However, under these conditions, EPCs function may be possibly influenced by additional factors that may be present in the culture. It has been well documented that, following stimulation, in addition to soluble factors, platelets are able to produce platelets microparticles. These microparticles carry an array of platelet-derived products including glycoprotein (GP) Ib,GPIIb/IIIa, P selectin, CXCR4 receptor, chemokines and various bioactive lipids, which can be transferred to recipient cells [29], [31]. Mause et al showed that platelet microparticles are able to enhance EPCs pro-angiogenic function and to modulate the phenotype of EPCs by transferring elements such as CXCR4 which originate from the microparticles [29]. Indeed, in the present study, the improvement in EPCs functional properties can be attributed not only to solvent factors but potentially also to platelet microparticles. However, although other elements are present in the culture, PDGF and FGF inhibition clearly attenuated the effect platelets exert on EPCs, and thus these factors appear to have a central role in the platelet-EPC interaction.

The definition and characterization of EPCs has been controversial. This controversy has recently been reinforced by several studies which indicated that EPCs might be mononuclear cells that obtain new characteristics as a result of culture exposure to microparticles from other elements in their microenvironment, such as platelets [29], [37]. Nevertheless, many studies have consistently defined and identified EPCs by the expression of CD133, CD34 and VEGFRII (or by a combination of these markers), and by their capacity to form typical colony forming units [13]–[18]. These studies also showed a significant correlation between the levels and function of EPCs, as defined in our study, and different cardiovascular disease states, as well as with adverse cardiovascular prognosis [13], [14], [15], [16], [17], [18], [38]. These observations support the notion that EPCs represent a group of progenitor cells which have an important impact and clinical role.

In the current study, we demonstrated that the positive effect of platelets on EPCs is mediated at least in part by secreted factors from platelets or from EPCs in response to platelets which are essential to this interaction. Our findings indicate that secreted factors from platelets, especially PDGF isoforms, have a crucial effect on EPCs functional properties and the development of an endothelial phenotype. Further study is needed in order to investigate EPCs phenotype in culture and identify additional key factors in this reaction.
